# Sexting, Self-esteem, and Social Media: A Comparison among Frequent, Occasional, and Non-sexting Italian Adolescent Girls

**DOI:** 10.11621/pir.2023.0401

**Published:** 2023-12-01

**Authors:** Valeria Verrastro, Valeria Saladino, Stefano Eleuteri, Nadia Barberis, Francesca Cuzzocrea

**Affiliations:** a University of Catanzaro, Italy; b University of Cassino and Southern Lazio, Italy; c Sapienza University of Rome, Italy

**Keywords:** sexting, adolescence, sexuality, self-esteem, social media

## Abstract

**Background:**

The phenomenon of sexting consists of sending and sharing sexual images, videos, or messages using smartphones and social networks. Sexting is spreading dangerously among adolescents who share private and explicit sexual content, ignoring the negative and risky consequences associated with this behavior. According to recent literature, sexting behavior is strongly related to the participants’ level of self-esteem and social media’s influence on them.

**Objective:**

Our study was a descriptive community-based study focused on the central-south^1^ Italian context, which aimed to investigate sexually risky behavior and the main motivations for sexting, and to compare differences in self-esteem and social media’s influence among three groups of Italian girls: non-sexters, occasional sexters, and frequent sexters.

**Design:**

Our research involved 569 Italian girls (14–19 years old) who completed the following questionnaires: the Health and Sexual Behavior Questionnaire; the Sexting Behavior Scale; the Multidimensional Self-Concept Scale; and the Sociocultural Attitudes Toward Appearance Questionnaire-3.

**Results:**

Our results showed that most participants sexted with their partner, and had a responsible attitude toward sexuality and kept sexual risk low. Moreover, frequent sexters had higher scores on social media’s influence relative to the internalization of the body image and model for beauty and athleticism, as well as lower levels of global, academic, and competence self-esteem.

**Conclusion:**

Our results could promote interventions in the school context in order to: a) improve awareness among youth about social media use, sexting, and safe sexuality; b) reduce the risk associated with sexting and the influence of social networks; and c) stimulate reflections by teachers and parents on adolescents’ sense of identity and self-esteem.

## Introduction

### Sexting: Theoretical frameworks

Teenagers’ daily lives are becoming more and more impacted by social media, continual connectedness, and virtual contact on the Internet and mobile devices. In this setting, intimate communication need not occur exclusively face-to-face, but also through various electronic media, including phone conversations, emails, texts, images, and videos.

The current discussion of adolescent sexting in both academic and popular circles has raised several concerns about how to reduce the negative psychological and legal consequences of this activity, and support adolescents’ sexual health and empowerment. Indeed, sexting — defined as the exchange of messages, videos, and photos with explicit sexual content through Smartphone applications (Semenzin & Bainotti, 2020; Ojeda et al., 2020; Campelli, 2021) — is commonly considered one of the main risks derived from the development of new technologies and social networks among teens (Saladino et al., 2020). In most cases, youth who sext are unaware of the possible negative consequences of this activity, including the risks of cyberbullying, blackmail, sexual violence, cyberstalking, revenge porn, sextortion, grooming, Pull a Pig, and suicide (Scherr, 2022; Hu, Clancy, & Klettke, 2023). Thus, sexting can lead to harmful outcomes and involve risks for the adolescent’s development (Cuccì et al., 2023).

In the Italian context, according to the National Observatory on Adolescents (2018), teens exchange intimate selfies — without clothes or with a sexual background with one’s partner — to friends in group chats without a real awareness of the possible consequences. From one online survey, it emerged that sexting is a practice routinely used by 6% of preteens age 11 to 13, of which seven out of 10 are girls. The numbers rise between the ages of 14–19. Indeed, one out of 10 adolescents sends sexually explicit content online. Girls are more at risk and are often also victims of revenge porn and cyberbullying derived from sexting, considering that 33% of the episodes of digital bullying are of a sexual nature. Along the same line, data from the Italian Federation of Scientific Sexology (FISS) has reported that 48% of adolescents practice sexting (Belli & Galletti, 2019).

Moreover, Migliorato et al. (2018) found that adolescents who engage in sexting have a lower perception of risk in their behavior and are more likely to drink, smoke, and engage in unprotected sex. This aspect could be associated with the tendency of adolescents to explore new situations and contexts in their attempts to define their autonomy and identity, while often not perceiving related risks (Saladino et al., 2020).

The negative narrative around sexting has occurred at the same time that the phenomenon seems to have become more normalized. Indeed, Mori et al. (2021) recently found that juvenile sexting is increasingly widespread compared to the past. This finding leads to the possible interpretation that early adolescents are exploring their sexuality in a protected context. From this standpoint, as posited by the concept of Positive Youth Development (Pistoni et al., 2023), sexting can be conceptualized as the modern manner that adolescents express their sexuality in the digital age (Yépez-Tito et al., 2020), as well as in intimate communication facilitated by technology.

Studies on how gender and sex correlate with sexting have found that girls are more likely than boys to be affected by peer pressure to engage in sexting behavior due to the fear of losing their partner (Van Ouytsel et al., 2017). In addition, sexting appears to be on the rise for those between adolescence and emerging adulthood (Choi et al., 2019).

A review of the literature shows the necessity of researchers promoting recommendations for caregivers and teachers to use in stimulating their children/students to think critically and to reflect on the consequences of their actions. This would include promoting more disclosure to adults and decreasing severe risks to the adolescents’ identity and autonomy within a positive developmental framework, thus enhancing the strengths and supporting the sexual well-being of young people in the digital age.

### The current research: Descriptive community-based study, goals, and hypothesis

Our study was a descriptive and community-based contribution focused on a sample of girls from the central-south area of Italy. We intended to draw attention to the phenomenon in a poorly explored context such as the central-south, with a sample exposed to the consequences of sexting, *i.e.*, girls. Our study aimed to explore sexting behavior with the following goals: a) describe the propensity of the sample to sext and their related motivations; and b) compare differences in self-esteem and the influence of social media among three groups of Italian girls, grouped according to their attitudes toward sexting behavior (non-sexters, occasional, and frequent).

We framed the following hypotheses: (H1) Participants who sext occasionally and frequently are more exposed to high-risk sexual behaviors; (H2a) Those who sext occasionally and frequently are more influenced by social media; and (H2b) Sexters have a lower level of self-esteem than those who do not sext. To the best of our knowledge, data on these questions are not currently available in the literature, and thus our study could contribute new knowledge on the subject.

Indeed, sexting spreads in several contexts and could have different motivations. Moreover, although sexting is a well-known and widespread phenomenon, there are poorly explored areas, such as the central-south of Italy, where there is a gap in the awareness about sexting-related sexual and developmental risks, especially among girls, who seems to be more affected by the consequences of this phenomenon. This gap could be related to a lack of sex education by the local institutions and families in sexual education. For this reason, our main aim was to investigate the frequency of sexting and its relationship with self-esteem and social media’s influence in this specific social group.

### Social media and body image among sexting girls

During adolescence, individuals develop a sense of autonomy and a personal perception of themselves based on external judgments and pressures derived from their social context, peer group, and social media (Eleuteri & Saladino, 2023).

Social media — such as television, magazines, website, and social networks — and social comparisons greatly influence our perception of our body image and promote an ideal of beauty which often leads to body dissatisfaction (Verrastro et al., 2020) and risky behavior (Eleuteri & Saladino, 2023). According to the literature, girls seem more affected by social media exposure on questions related to aesthetical values and body satisfaction.

For instance, the Public Use Microdata Files of the Canadian Community Health Survey (Statistics Canada, 2012; Carter et al., 2017) involved 130,000 Canadian girls (12–29 years) in investigating the association between their body dissatisfaction and amount of time online. They found that participants who spent more than 20 hours per week online (especially girls 11-, 13-, and 15-year-old) showed higher levels of body dissatisfaction than the group with the least Internet use (none/ < 1 hour). According to the authors, time spent online leads to higher exposure to the sociocultural influences that confirm the importance of the beautiful physical appearance as a central component of a girl’s identity. This exposure could be linked to the internalization of an unattained female beauty model and thus increased body dissatisfaction (Speno & Aubrey, 2019).

Sexting girls might aspire to achieve the perfect appearance proposed by social media, and search for gratification about their body image, due to their internalization of proposed models. This idealization produces a conflict, leading to lower body satisfaction (Dully et al., 2023). Mostly, girls tend to manipulate their pictures by presenting themselves as perfect and beautiful, and the more they modify the images offered on the web, the more they lose the ability to interpret reality about their aesthetic identity and that of others, basing themselves only on social comparisons.

This interpretation is consistent with the study by Kleemans et al. (2018), who investigated the negative effect on the body image of manipulated Instagram* photos, and the role played by the tendency to rely on social comparison. The authors randomly exposed 72 girls of 14–18 years to 10 original Instagram^*^ photos, and 72 girls of the same age to 10 manipulated Instagram* photos. The results showed that the group exposed to manipulated photos reported lower body satisfaction, especially among girls with a higher tendency to rely on social comparison. Thus, social comparison mediates the perception of body image, leading girls to manipulate their photos to be accepted by the community, and thus feel less discomfort toward their bodies.

Mostly, girls with a higher tendency to rely on social comparisons do not notice that pictures are reshaped or manipulated, thinking that they are real because of their systematic exposure to the ideal of beauty. Moreover, sexting girls seem to be more sensitive to this exposure due to the goal of their pictures. For example, girls of 6-7 years post explicit sexual videos on the video-sharing app musical.ly and manipulate and retouch them, imitating their popular models and developing an obsession about their appearance and sexualization (Rondino, 2018).

Thus, this focus on the ideal body and the perception of aesthetical satisfaction impact sexual development and can increase the possibility of sexting. Howard et al. (2019) investigated this connection and found that the group with a negative perception of their appearance reported feeling higher pressure to sext, because in sending and receiving sexts, their body dissatisfaction decreased. This phenomenon could potentially represent a rewarding experience for sexters, who are more vulnerable to being victimized through coercion or manipulation due to their external motivation in doing sexting and in being more exposed to other comments. Bianchi et al. (2017) confirmed these results, finding that people who compare their bodies to the cultural and social standard of beauty are more likely to sext in order to receive positive reinforcement.

Based on the cited results, our study aimed to elaborate on the hypothesized association between the frequency of sexting, the attitude toward sexting, social media’s influence on the internalization of the sexters’ aesthetic values, and the self-esteem of the specific target group.

### Self-esteem, sexting related motivations, and sexuality

Self-esteem is a socio-psychological construct derived from social interactions. Individuals develop self-esteem through interpersonal relationships from childhood to adulthood, experimenting with different contexts and roles. Teens are more likely to be influenced by peers in developing their sense of self-esteem, according to the scientific evidence (Antonopoulou, Chaidemenou, & Kouvava, 2019). One of the most emphasized concepts in studies of self-esteem among youth is their need to belong to a group and the influence that low self-esteem has on some risky behaviors, such as substance abuse, sexually risky behavior, problematic internet and social media use, and sexting (Wang et al., 2017; Saladino et al., 2020).

For the most part, adolescents with low self-esteem experience a feeling of inferiority, self-dissatisfaction, and a higher need for peer approval. Specifically, regarding body perception and sexting, research has shown that those less satisfied with their body and less confident in emotional and sexual relationships tend to sext in order to receive social gratification (Howard et al., 2019; Howard et al., 2021).

In line with these results, Wachs et al. (2017) highlighted in their study that increased self-esteem and self-control lead to decreased sexting.

This result seems to be more evident among adolescents than adults. Indeed, there are no differences in the level of sexting (non-sexters, sexters who send and receive, and sexters who receive), and the levels of self-esteem among young adults (Sharma et al., 2019). One possible explanation is related to the lower need for approval and the different impact of social media among adults, who have already developed their sense of identity, and often use sexting within their intimate relationship, and are more aware of the consequences (Jeanfreau et al., 2019).

Thus, self-esteem plays a protective role in sexting, and in lessening the influence of social media, especially among adolescents with lower self-esteem, who are more sensitive to the aesthetical ideal and the information provided by media (Dully et al., 2023). Indeed, sexting is associated with the desire to be perceived as beautiful and popular (Eleuteri et al., 2017). Many teens have not experienced sexual intercourse and are afraid because they feel uncomfortable with their aesthetical aspect. Online contact, mediated by technological devices, can increase self-awareness and security (Howard et al., 2019). Sexting might be the first approach to sexuality among many adolescents or a part of a couple’s sexuality (Kosenko et al., 2017).

From this standpoint, the association between sexting and sexual risks is not linear. Indeed, Davis et al. (2016) see motivations for sexting as a way of discriminating between healthy sexual enrichment within a stable relationship, and a way to captivate new and casual partners, an attitude that may affect sexual health.

Moreover, some individuals sext for intra-individual motivations, such as exploring sexuality, flirting, or having fun (Bianchi et al., 2016, 2017), sexual empowerment, or personal expression (Liong & Cheng, 2019); other individuals sext for extra-individual motivations, such as looking for positive reinforcement for their body image and drawing attention to themselves. Most individuals seem to sext for extra-individual reasons (Bianchi et al., 2016), especially to receive feedback on their aesthetical aspect; this leads to negative consequences.

We were interested in investigating sexting-related motivations — analyzed qualitatively and descriptively in our protocol — in addition to the relationship between self-esteem and the influence of social media on the frequency of sexting. Although evaluating reasons for sexting was not the focus of our work, the literature indicates that motivation affects the greater or lesser probability of risks associated with sexuality.

## Methods and Measurements

### Participants

Our sample was composed of 569 girls (mean age = 16.93; SD = 1.375; age range: 14– 19) recruited from high schools in the central-south area of Italy.

The researchers explained the aims and scope of the research to the adolescents and their parents, and obtained informed consent signed by the parents, and authorization from the school Director. The study was conducted following the standards set by the Declaration of Helsinki and was approved by the Institutional Review Board of the University of Cassino and Southern Lazio (Italy).

### Procedures

The questionnaires were administered during regular school hours under the supervision of the teachers and the research team. This process took approximately 60 minutes. The participants were informed of the complete anonymity of the information they were about to provide. Participants were aware that they could stop completing the questionnaires at any time and request that their data not be used.

### Measurements

*The Questionnaire on Health and Sexual Behavior* (University of Cassino and Southern Lazio) is a self-report developed for this study by the University of Cassino and Southern Lazio research team. The questionnaire is composed of eight dichotomous items which explore aspects of sexual behavior (sexual intercourse, age of sexual intercourse, relationship status, information on contraception, morning-after pill use, sexually transmitted diseases, pregnancies, and abortions). This questionnaire aims to collect general data on sexual behavior to explore adolescent sexual awareness and experience.

*The Sexting Behavior Scale* (SBS) (Dir et al., 2013; Morelli et al., 2016) is a self-report tool which investigates the presence or absence of sexting activities and the motivations related to it. The first part contains eight items on a 5-point Likert scale, which measure the frequency of sexting behavior and the means used (*e.g.*, How often have you sent erotic images via Facebook*?). The second part aims to identify the reasons and the risk factors associated with sexting (*e.g.*, I practice sexting when I use alcohol; I practice sexting because I want to have sex). The questionnaire has a Cronbach alpha ranging from .80 to .89.

*The Multidimensional Self-Concept Scale* (Bracken & Howell, 1991; Bracken, 2003) is a self-report characterized by 150 items which assess self-concept and self-esteem on a 4-point Likert scale in six categories: 1) social (interpersonal relationships); 2) academic (school perceived success); 3) affect (emotionality); 4) family (familial relationships); 5) physical (body image); and 6) competence (control over one’s environment). A lower score indicates lower self-esteem. The questionnaire has a Cronbach alpha ranging from .70 to .80.

*The SATAQ-3-Sociocultural Attitudes Towards Appearance Questionnaire-3* (Thompson et al., 2004; Stefanile et al. 2011) is a self-report tool which measures the influence of society and the media on body perception and self-image. It is composed of 30 items, evaluated on a 4-point Likert scale, divided into four subscales: 1) general internalization of the body and beauty proposed by the media (Internalization-General); 2) internalization of an athletic physical model (Internalization-Athlete); 3) pressures from the outside to coincide with the stereotype (Pressures); and 4) perception of the media as a credible and essential source of information regarding fashion and beauty (Information). High scores indicate greater adherence to the stereotype. The questionnaire has a Cronbach alpha ranging from .80 to .89.

### Statistical Analysis

The data were analyzed using the Statistical Package for Social Sciences (Version 26.0, SPSS Inc., Armonk) (IBM Corp. Released, 2019).

Descriptive analysis was used to show the youth’s attitudes toward the use of contraceptive methods, the risks associated with sexuality, their main motivation, and the context of sexting behavior of the sample.

One-way ANOVAs were used with Fisher post-hoc tests to examine differences among the three groups of girls in self-esteem and social media influence.

Cohen’s d effect size (ES) was utilized to calculate the magnitude of the differences among groups.

The sample was divided according to the girls’ attitudes toward sexting, based on their scores on the Sexting Behavior Scale (SBS) (Dir et al., 2013; Morelli et al. 2016). The questionnaire has a minimum score of 8 and a maximum of 40. According to the point distributions identified by the authors (Dir et al., 2013) and to the distribution used by Verrastro et al. (2017), minimum, medium, and maximum scores were identified, establishing three different paths of behavior: no sexting (8 points); occasional sexting (9-12 points); and frequent sexting (>13 points).

## Results

### Participants’ profile

Our sample was composed of 71.5% sexters, of whom 37.8% sexted occasionally and 33.7% sexted frequently.

When we evaluated the group of participants who sext regarding their attitude towards sexuality, 57.5% said they had had sexual intercourse, and 56.4% did not use contraceptive methods. Moreover, 92.2% of the sample did not use the morning-after pill. According to these results, on one hand, it seems that the girls ignored the risks associated with unprotected sexual intercourse and tend to engage in sexual intercourse.

However, these results should be interpreted in light of the girls’ attitudes toward sexting and their related motivations (as reported in *Table 1*). Indeed, most of the participants sexted with their partner with the aim of engaging in a physical sexual relationship. Although most of them did not use contraceptives, or were not fully aware of the risks associated with their behavior, 99.5% never referred to having contracted sexually transmitted infections; never had to go through a pregnancy (98.3%); and had not ever voluntarily terminated a pregnancy (99.3%). These results confirm a flexible interpretation relative to sexual risks and sexting (see Discussion).

**Table 1:** Motivation and context of sexting behavior among participants

Question	Answer	Percentage
Who do you sext with?	Partner	74.4%
When do you sext?	When I am at home	24.7%
Why do you sext?	I would like to have sex	45%

### Sexting, social media, and self-esteem: differences among girls

We have conducted two one-way ANOVAs with Fisher’s LSD post-hoc tests to compare the three groups of participants (non-sexters, occasional sexters, and frequent sexters) on the degree to which they were influenced by social media (*Table 2*) and on their self-esteem (*Table 3*).

**Table 2:** ANOVA on social media and sexting variable between three groups, mean and standard deviations

	Group			*p*	*F*-value
	Non-sexters	Occasional sexters	Frequent sexters		
Intern-General	22.51(5.55)	24.72(8.13)	26.38(8.70)	< .001	9.74
Intern-Athlete	12.24(3.48)	12.31(3.61)	13.25(3.95)	.020	3.95
Pressures	20.88(2.75)	21.02(2.14)	21.02(2.21)	.832	.184
Information	25.30(5.83)	26.56(5.80)	27.41(6.53)	.006	5.09

**Table 3:** ANOVA on self-esteem and sexting variable between three groups, mean and standard deviation

	Group			*p*	*F-*value
	Non-sexters	Occasional sexters	Frequent sexters		
Social	72.26(8.63)	72.23 (9.29)	71.77(11.34)	.871	.138
Academic	68.57(6.72)	67.34(7.14)	65.94(7.07)	.002	6.23
Affect	64.62(3.88)	64.90(3.94)	64.13(4.27)	.159	1.84
Family	60.83(2.72)	61.25(3.07)	61.24(3.16)	.353	1.04
Physical	32.50(3.17)	32.15(3.08)	32.77(2.83)	.123	2.10
Competence	69.10(7.35)	68.56(8.09)	67.02(8.28)	.042	3.18
Global score	368.53(18.26)	366.34(20.39)	362.60(22.03)	.038	3.28

*Table 2* shows the means and standard deviations of the first ANOVA. Regarding social media influence, there were significant differences between the three groups on the following subscales: Internalization-General, Internalization-Athlete, and Information.

The Fisher’s LSD post-hoc test revealed a significant difference between the first and third group of participants on Internalization-General (*p* < .001; ES = 0.530), Internalization-Athlete (*p = .*047; ES = .271), and Information (*p* = .005; ES = 0.341), and between the second and third group on Internalization-Athlete (*p* = .046; ES = 0.248), while no differences were found for Pressures.

*Table 3* shows the means and standard deviations of the second one-way ANOVA regarding global self-esteem and its six sub-scores — social, academic, affect, family, physical, and competence. There is a statistically significant difference between these groups in the academic area, competence area, and in global self-esteem. A Fisher’s LSD post-hoc test revealed a significant difference between the first and the third group of participants (*p* < .001; ES = .381) on the global score, the second and the third group in academic area (*p* = .044*;* ES = .197), and the first and the third group of participants in competence area (*p = .*017; ES = 0.266). Moreover, global self-esteem differed between the first and the third group (*p* = .012; ES = .293).

## Discussion

### Principal findings

Our study aimed to analyze the phenomenon of sexting in a group of Italian teenage girls (14-19 years). Self-esteem and the influence of social media have been examined as variables that could be related to sexting behavior. The sample was divided into three groups, according to their involvement with sexting: frequent, occasional, and non-sexters. A total of 71.5% of the participants sexted, of which 37.8% sexted occasionally and 33.7% sexted frequently.

From the descriptive analysis, we found that most of the participants who sexted had had sexual intercourse (57.5%) and did not use contraceptive methods (56.4%). In our sample, the girls tended to sext with their partners when they were at home due to the desire to engage in sexual intercourse, thus showing an intra-individual motivation (Bianchi et al., 2016, 2017; Dir et al., 2013). According to some studies, intra-individual reasons for sexting protect individuals from negative consequences related to sexual risks (Souza & Lordello, 2020; Morelli et al., 2016).

Most participants reported having no sexually transmitted venereal diseases, pregnancies, or pregnancy interruptions. These results partially disconfirm our hypothesis, according to which sexters would have a higher percentage of high-risk sexual behavior (H1). However, they are in line with the literature (Dir et al., 2013; Davis, et al., 2016; Bianchi et al., 2016, 2017; Liong & Cheng, 2019). Indeed, according to the framework of the Positive Youth Development Approach (PYD) and to the recent perception of sexting as a path of behavior which describes adolescents’ development, youths could explore their sexuality through sexting and improve their understanding of their feelings related to their body image (Pistoni et al., 2023), using sexting to approach sexuality for the first time, or to explore their sexuality within their relationship (Kosenko et al., 2017).

Regarding hypothesis two (H2a), our results showed significant differences between the groups, especially between frequent sexters and the non-sexting group, on the influence of social media. Frequent sexters showed higher levels of social media’s influence relative to the subscales of the internalization of the body and beauty images proposed by social media (Internalization-General), the internalization of the athletic model (Internalization-Athlete), and the perception of the media as a credible and essential source of information regarding fashion and beauty (Information). These results confirm our hypothesis and are in line with recent literature.

We know from scientific evidence that social media have a higher influence on girls’ perception of their bodies (Saladino et al., 2020) and sexual identity (Eleuteri et al., 2017, Verrastro et al., 2017) than on boys. Indeed, girls develop body dissatisfaction due to social comparison with the ideal of beauty and athletic models proposed by television, magazine, and social networks (Tamarit et al., 2021). During the last few years, sexting has become one of the most widespread ways of exploring sexuality, and adolescents spend an increased amount of time online.

Increased time online leads to higher exposure to sociocultural influences related to female esthetical values and the internalization of female beauty models (Speno & Aubrey, 2019). Websites, social networks, and all online news represent a credible source of information for teens (Rawat et al., 2020), who often avoid asking for advice or communicating with adults, and prefer to rely on websites and social networks to satisfy their needs and address their questions on how to lose weight, how sexuality works, and which are the most popular beauty and fashion models (Carter et al., 2017). In particular, girls are more exposed than boys to the influence of social media and manipulated photos which represent a distortion of reality, provoking a constant comparison with pictures of “perfect” bodies, and a general discomfort with their own (Van Ouytsel et al., 2017).

Moreover, this content is often explicitly sexual and tends to objectify the female body. An example could be musical.ly, a platform used most by girls from 6-7 years to watch explicit sexual videos, imitating popular models (Rondino, 2018). However, we found no differences between the three groups in the pressure from the outside to coincide with the stereotype (Pressures). This result is controversial since it contrasts with the literature’s emphasis on the role of pressure from peers and social media in promoting sexting (Barrense-Dias et al., 2017; van Oosten & Vandenbosch, 2017).

The tendency to sext is also related to the sexters’ level of self-esteem. Our results underlined differences between the frequent sexter and non-sexter groups in their global self-esteem score. Frequent sexters showed lower global self-esteem, in line with recent literature. Adolescents with low self-esteem could have a lower self-satisfaction and confidence in a sexual relationship. Thus, they could be more likely to use sexting to compensate for their need for approval, to explore their sexuality mediated by a screen or to receive social gratification (Howard et al. 2019; Howard et al., 2021). Self-esteem has a protective role, especially among vulnerable teens, vis-a-vis social media’s influence and sexting. Our sample seemed to show that girls sext for a personal motivation related to the desire to engage in a sexual intercourse with their partner. This aspect could indicate that they could be less at risk of the negative consequences of sexting due to the exclusive relationship with their partner. However, longitudinal studies are needed to evaluate and monitor the long-term impact of sexting. One less studied aspect is the influence of sexting behavior on self-esteem relative to other spheres of an individual’s life, such as those investigated by the Multidimensional Self-Concept Scale (Bracken & Howell, 1991; Bracken, 2003). This scale is a self-report that assesses self-esteem in six areas: 1) social (interpersonal relationships); 2) academic (school perceived success); 3) affect (emotionality); 4) family (familial relationships); 5) physical (body image); and 6) competence (control over the environment).

Previous research (Wachs et al., 2017) investigated global self-esteem only. To fill in this gap, we focused on more than one area and got unexpected results: the differences between non-sexters and frequent sexter groups in self-esteem were related to the academic and competence dimensions. In addition to their lower level of global self-esteem, the frequent sexter group reported lower academic and competence self-esteem. Academic self-esteem is associated with a positive perception of one’s self, school success, and relationships with classmates (Zheng et al., 2020). Indeed, students with a good perception of themselves are more likely to achieve competence and obtain better test scores at school. Also, during adolescence, individuals typically base their self-esteem and behavior on external judgments and pressures derived from their social context, peer group, and social media (Eleuteri et al., 2017). School is a social environment in which to establish relationships, develop perceptions of themselves and others, and learn such behaviors.

Similarly, the sense of competence can be associated with agency, the perception one can deal effectively with events to control the environment and feel appreciated by others. Sexting for some individuals is a way to affirm their identity and empowerment (Liong & Cheng, 2019). During adolescence, the sense of self-confidence generally derives from others’ feedback, and often youth feel unable to manage their lives. This feeling can lead them to create an idealized image that makes them feel more powerful, such as sexualized photos, which increase their social gratification (Howard et al., 2019).

Another aspect is the importance of self-esteem as related to body image; however, this was not significant in our sample. According to the literature, low self-esteem is associated with body self-esteem issues and an increased susceptibility to influence by social media platforms (Peris et al., 2019), due to adolescents’ need to receive validation to compensate for their low body satisfaction; they thus become more vulnerable online (Longobardi et al., 2021).

These findings need to be thoroughly researched in the future, in order to investigate sexters’ self-esteem in the academic and competence areas, and to evaluate the crucial role of body self-esteem in social media influence and sexting behavior.

## Conclusion

Our study aimed to explore the possible relationship between self-esteem and the influence of social media in an seldom-study sample, girls of the central south of Italy. The results are not generalizable due to the research design and the limited number of the participants. However, the data provide some insights into the relationship between sexting and its motivation, and on the role of self-esteem in the relationship between sexting frequency and social media influence.

This study suggested that sexting could be a way for couples to explore their sexuality or to establish sexual empowerment and identity in teens. The relationship with a partner, intra-individual motivations, and high self-esteem are possible protective factors against the negative consequences of sexting.

Thus, the most relevant aspect to consider is the sexter’s self-awareness and the real motivations for their behavior. Adolescents who sext in order to receive social gratification are more likely to experience body dissatisfaction and be negatively affected by the ideal of beauty and perfection on social media. They can also be victims of sexual coercion, sextortion, cyberbullying, cyberstalking, and sexual harassment. On the other hand, sexting used with more awareness could reduce developmental and sexual risks. This approach, in line with the recent studies and reviews on the topic, challenges the conclusion that the sexting phenomenon is solely negative, perceiving it as a behavior which, if carried out in a consensual manner and without harmful intent, might be a component of an individual’s healthy relational and sexual life.

These results provided suggestions for future elaboration on the individual, educational, familial, and environmental levels. For instance, one recommendation for clinicians could be an evaluation of Italian girls’ awareness of the possible consequences and risks associated with sexting as part of sexual education programs in the schools, which are not promoted in Italy, as they should be. Moreover, data on social media’s influence and impact on girls’ self-esteem could be used for support of teens and parents (Gugliandolo et al., 2019), deepening the role of academic success and competence in the environment and informing them about a safer use of social media and sexting.

Furthermore, there are some key points to investigate, such as the study of motivations related to sexting; the long-term sexual and behavioral effects of sexting; the extension of the research to a male sample, considering the types of sexters, those who send, those who receive, those who send and receive photos, videos or messages; the investigation of the connection between self-esteem and social media’s influence; and the study of the possible mediating role of self-esteem in choosing to sext.

Finally, actions are needed at policy levels to ensure that websites and social networks convey more realistic and healthy values that inspire girls and young women to take care of themselves and not to search for unrealistic perfection, by promoting different and more realistic representations of the female aesthetic model.

## Limitations

One of the limitations of this study was the failure to investigate such factors as the youth’s level of communication, family climate, parental attachment, and relationship with peers, all of which can impact youth behavior. As reported by some research on this topic (Punyanunt-Carter, 2008; Gumede et al., 2017; Haverfield & Theiss, 2017) a positive relationship with parents and belonging to a supportive peer group can protect young people from the consequences of risky behavior, including sexting (Campbell & Park, 2014; Bianchi et al., 2019; Burén et al., 2021).

A second limitation concerns the sole focus on a female sample; a deeper investigation of boys’ behavior relative to sexting is needed, as there is little research on it, especially among Italians. Moreover, it is not possible to determine the direction of causality due to the descriptive and cross-sectional design we used to carry out the research. Our data collection was based exclusively on the use of self-report, thus increasing the interpretation bias of the results. Finally, the classification of the three groups of sexters was based on a scale. Therefore, future studies should consider a longitudinal design with mixed methods to increase the accuracy of the results.

## Footnotes

* — Activities of Meta Platforms Inc. (Facebook and Instagram), and the X Corp. (as the successor of Twitter Inc.), are prohibited in the Russian Federation.
